# Supplementary material for: A polyamine acetyltransferase regulates the motility and biofilm formation of Acinetobacter baumannii
Source: Nat Commun. 2023 Jun 14;14:3531. doi: 10.1038/s41467-023-39316-5 (PMC10267138; doi:10.1038/s41467-023-39316-5)
Supplement: Supplementary file 1 — Supplementary Information [file 41467_2023_39316_MOESM1_ESM.pdf]

Supplementary Information

A polyamine acetyltransferase regulates the motility and biofilm formation of *Acinetobacter baumannii*

Julija Armalytė, Albinas Čepauskas, Gabija Šakalytė, Julius Martinkus, Jūratė Skerniškytė, Chloé Martens, Edita Sužiedėlienė, Abel Garcia-Pino, Dukas Jurėnas

Supplementary Figures and Captions

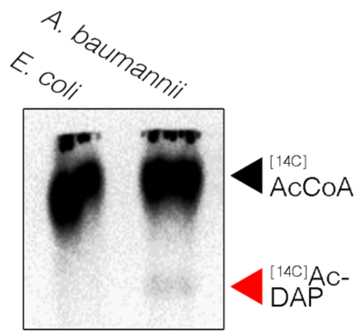

Supplementary Fig. S1 | Detection of substrates of Dpa enzyme in cellular extracts. (a) The natural substrate of the Dpa enzyme is present in *A. baumannii* cellular extract, but not in *E. coli*. The cellular extracts were prepared from late exponential phase bacteria as described in the methods section.

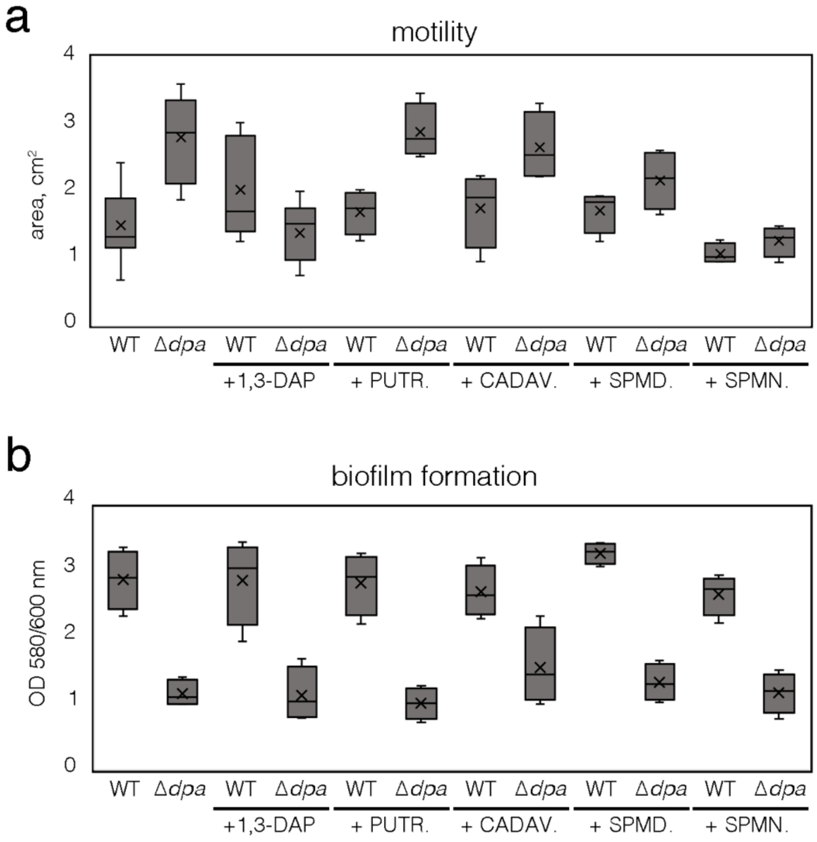

Supplementary Fig. S2 | Effect of polyamines on motility and biofilm formation. (a) The twitching motility of A. *baumannii*. Bacterial growth zone (in cm^2) around the inoculation site of bacteria were grown with or without 0.1 mM of different polyamines added to the media. The first four bar graphs correspond to the same data presented in Figure 1 b and are shown here for the reference. Experiments were executed at the same time for all the conditions shown. (b) Biofilm formation of *A. baumannii* and its *dpa* mutant in presence of different polyamines. Biofilms were allowed to grow for 18 h at 37 ° C, planktonic cells were removed and biofilms were quantified by crystal violet staining. Values were normalized to the optical density of the planktonic bacteria recovered from the well. Top and bottom of the box plot whiskers show maximum and minimum values, top and bottom of the box - 75th percentile and 25th percentile and line through the box with x markers show the median and the mean of the sample respectively. Each condition has been tested four times in biologically independent experiments, first four conditions of motility have been tested nine times. Source data is provided at the end of the Supplementary information file.

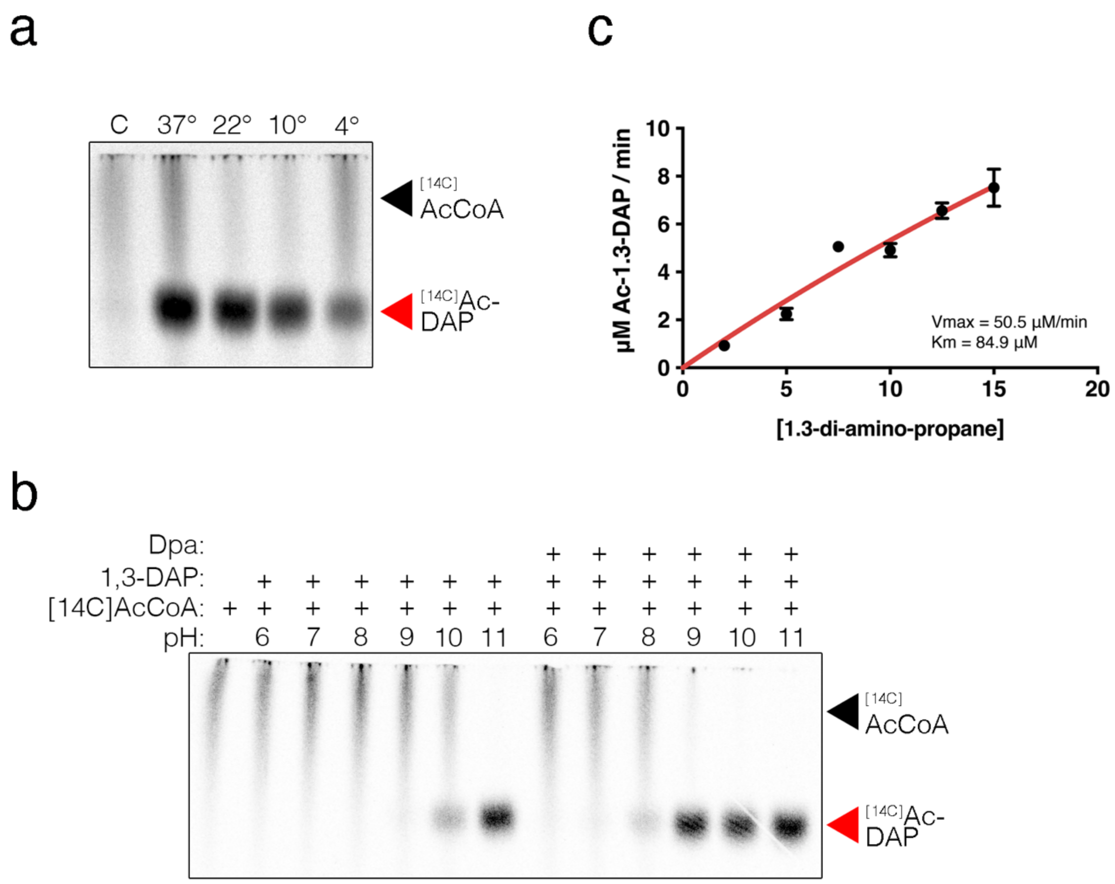

Supplementary Fig. S3 | Dpa kinetics. (a) Temperature dependent and (b) pH dependent acetylation of 1,3-DAP by Dpa, as compared to the spontaneous acetylation. (c) kinetics of 1,3-DAP acetylation by Dpa; Vmax and Km are indicated. Each reaction was performed three times over independent experiments. Source data is provided at the end of the Supplementary information file.

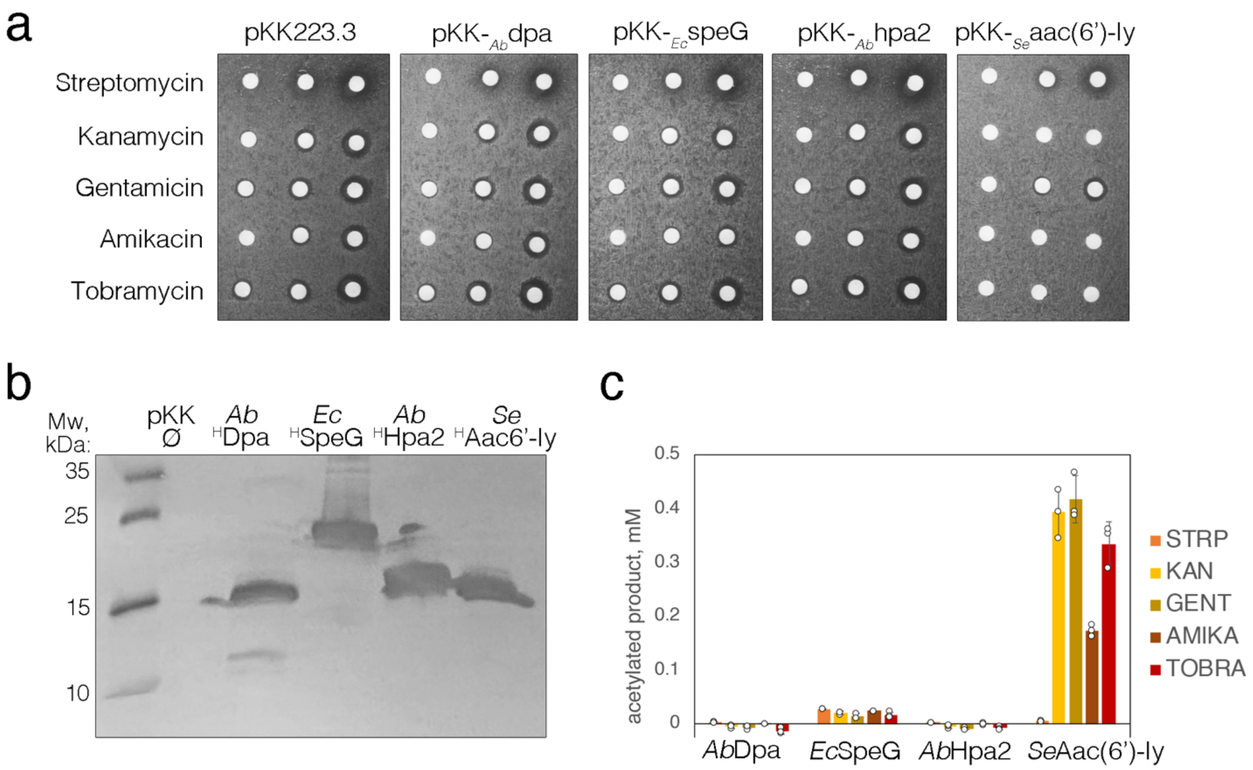

Supplementary Fig. S4 | Aminoglycoside antibiotic resistance and acetylation. (a) *E. coli* cells transformed with pKK223.3 vectors carrying difference GNAT acetyltransferases (*A. baumannii* Dpa, *E. coli* SpeG, *A. baumannii* Hpa2 or *S. enterica* Aac(6')-I 5y) were grown to mid-exponential phase, acetyltransferase production was induced with 0.5 mM IPTG for 2 hours and pre-induced bacteria were spread on solid LB agar containing 0.5 mM IPTG. Whatman discs soaked in series of 2-fold dilution of antibiotics (final concentrations of 256, 128 and 64 µg/mL of Streptomycin; 128, 64 and 32 µg/mL of Kanamycin; 64, 32 and 16 µg/mL of Gentamicin, Amikacin or Tobramycin) were then deposited on the bacterial loans. Plates were incubated overnight at 37 °C and zones of inhibition were visualized. (b) Expression of acetyltransferases was controlled by western-blot against his-tag. Experiments were performed four times independently; representative pictures and western blot are shown. (c) *In vitro* acetylation of 5 mM of antibiotics in presence of 0.5 mM acCoA, with 2 µM of enzyme at 30 °C for 30 min. Acetylation was quantified with DTNB reagent as described in the methods section. Each reaction was performed three times over independent experiments. Source data are provided at the end of the Supplementary information file.

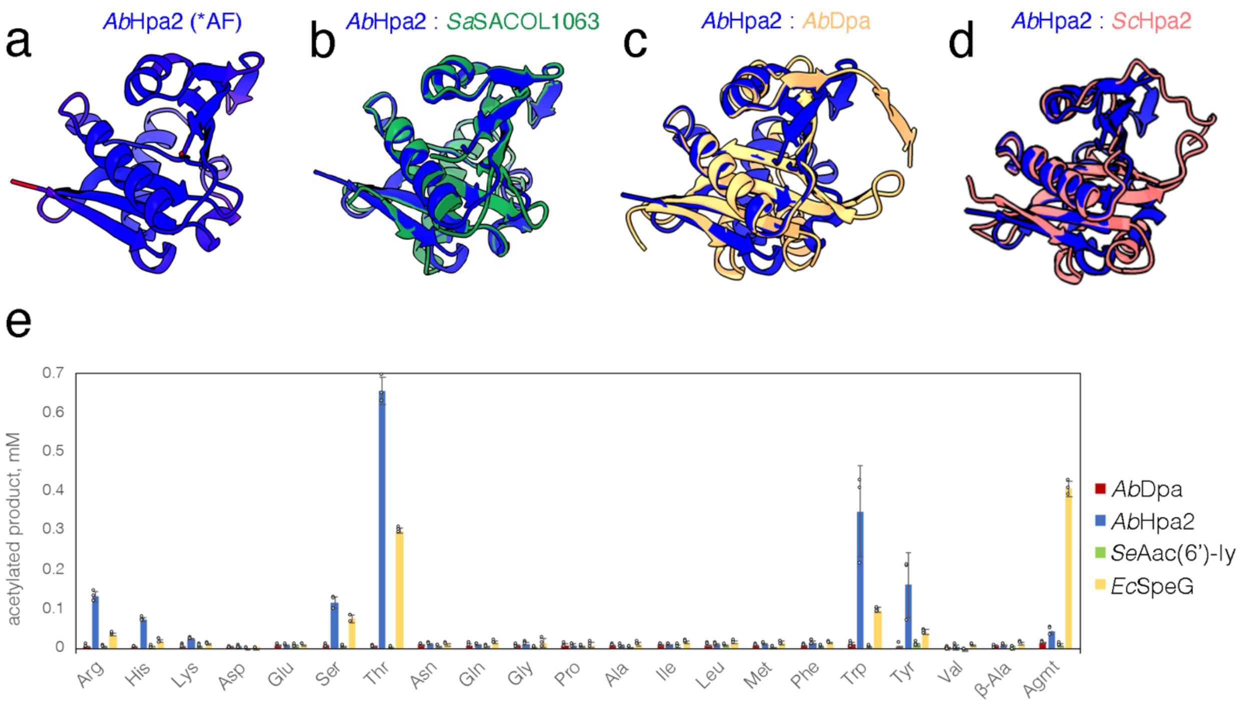

Supplementary Fig. S5 | *A. baumannii* Hpa2 is homologous to S. aureus SACOL1063 and acetylates a subset of amino acids. (a) AlphaFold2 model of *A. baumannii* Hpa2 colored by confidence from blue (100% confident) to red (not confident). TM-score (pTM = 0.9) was obtained for the best-ranked model shown here. (b) Structure alignment with closest structural homologue *S. aureus* SACOL1063 (PDB: 5JPH), based on DALI sever (Z score 24.0, rmsd 1.2). Alignment was executed with Chimera; *R.m.s.d.* between 115 pruned atom pairs is 0.734 Å; across all 137 pairs – 2.495 Å. (c) Structure alignment of *A. baumannii* Hpa2 model and Dpa; *R.m.s.d.* between 52 pruned atom pairs is 0.752 Å; across all 130 pairs - 8.271 Å. (d). Alignment of *Ab*Hpa2 with yeast Hpa2; *R.m.s.d.* between 48 pruned atom pairs is 0.928 Å and across all 127 pairs is 7.111 Å. (e) *In vitro* acetylation of 5 mM different amino acids in presence of 0.5 mM acCoA, with 2 µM of the enzyme at 30 °C for 30 min. Acetylation was quantified with DTNB reagent as described in the methods section. Cysteine was omitted due to reactivity with DTNB. In addition to canonical amino acids, the β-alanine (β-Ala) and polyamine precursor agmatine (Agmt) were included. Each reaction was performed three times over independent experiments. Source data is provided at the end of the Supplementary information file.

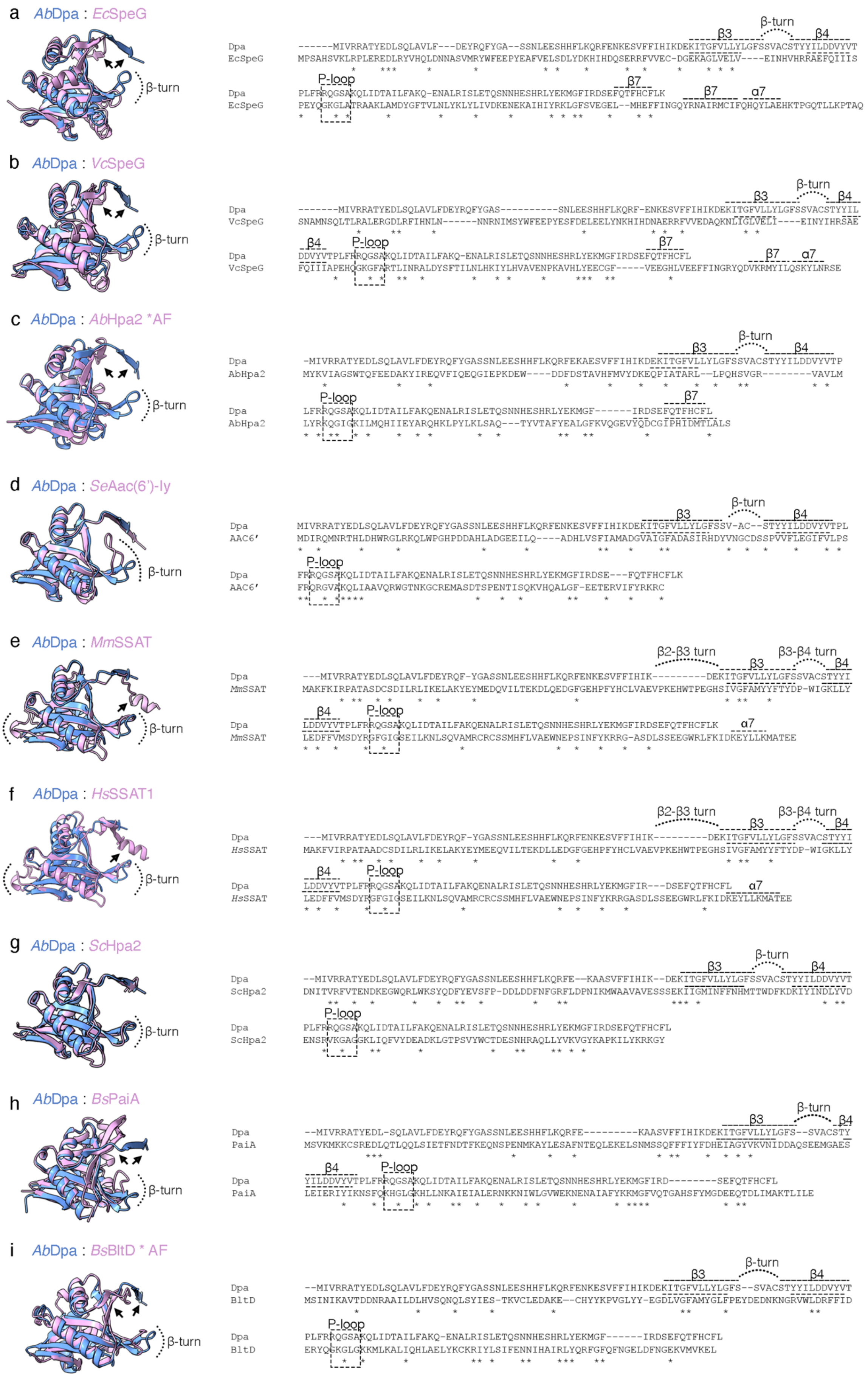
a AbDpa : EcSpeG
b AbDpa : VcSpeG
c AbDpa : AbHpa2 *AF
d AbDpa : SeAac(6')-Iy
e AbDpa : MmSSAT
f AbDpa : HsSSAT1
g AbDpa : ScHpa2
h AbDpa : BsPaiA
i AbDpa : BsBltD * AF
β-turn
β3
β4
β7
α7
P-loop
β2-β3 turn
β3-β4 turn
Dpa
EcSpeG
VcSpeG
AbHpa2
AAC6'
MmSSAT
HsSSAT
ScHpa2
PaiA
BltD

Supplementary Fig. S6 | Structure and sequence alignments of different GNAT acetyltransferases reported to actylate polyamines. (a) *A. baumannii* Dpa monomer alignment with *E. coli* SpeG (PDB:4R9M; *r.m.s.d.* = 6.88), (b) with *V. cholerae* SpeG (PDB:4JLY, *r.m.s.d.* = 4.996), (c) with *A. baumannii* Hpa2 (AlphaFold2 model, ptm = 0.9; *r.m.s.d.* = 8.271), (d) with *S. enterica* Aac(6')-Iy (PDB:1S5K; *r.m.s.d.* = 4.077), (e) with mouse SSAT (PDB:3BJ7; *r.m.s.d.* = 3.79), (f) with human SSAT (PDB:2B5G; *r.m.s.d.* = 3.649), (g) with yeast Hpa2 (PDB: 1QSO; *r.m.s.d* = 2.588.), (h) with *B. subtilis* PaiA (PDB: 1TIQ; *r.m.s.d.* = 7.289) and (i) with *B. subtilis* BltD (AlphaFold2 model, ptm= 0.85; *r.m.s.d.* = 6.687). Conserved P-loop structure that coordinates acCoA binding is boxed, and variable β-turn regions and C-terminal elements are indicated.

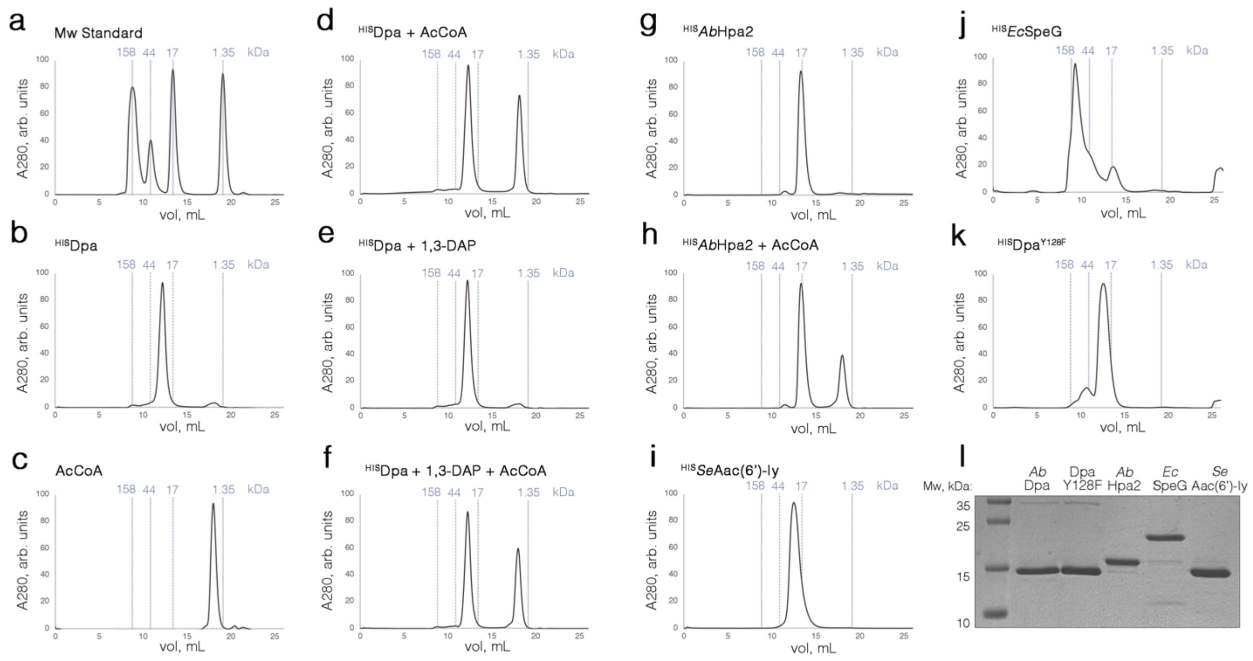

Supplementary Fig. S7 | Analytical size exclusion chromatography. (a) Molecular weight standards (BioRad) migrated on Superdex75 1030 increase column in the same running conditions as all the following samples. (b) Analytic SEC of Dpa enzyme alone, (c) acetyl-Coenzyme A, (d) Dpa premixed with 5-fold molar excess of acetyl-Coenzyme A or (e) 1,3-DAP or (f) both acCoA and 1,3-DAP an hour before the run. (g) Analytic SEC of *A. baumannii* Hpa2 alone or (h) premixed

with 5-fold molar excess of acCoaA. (i) Analytic SEC of *S. enterica* Aac(6')-Iy, (j) of *E. coli* SpeG, (k) of *A. baumannii* DpaY128F mutant. (l) SDS-PAGE gel analysis of proteins used for analytic SEC analysis. Protein purifications have been performed three times for all proteins and more than five times for Dpa and its mutated derivative, representative SDS-PAGE migration is shown.

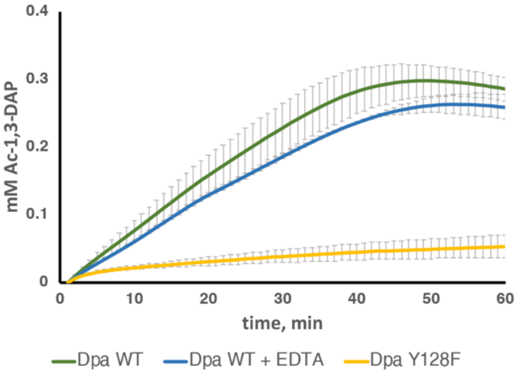

Supplementary Fig. S8 | 1,3-DAP acetylation kinetics. Dpa wild-type (WT), mutated Dpa_{Y128F} enzyme or Dpa treated with 50 mM EDTA overnight and re-purified by SEC, were used at a concentration of 2 µM with 10 mM of 1,3-DAP, 0.5 mM acCoA and 0.2 mM of DTNB. 10 mM EDTA was additionally added to the reaction of EDTA-pre-treated enzyme (Dpa WT + EDTA condition). Reactions were measured with microplate reader every 2 minutes for 1 hour. The average of three measurements is plotted; error bars show standard deviations. Each reaction was performed three times over independent experiments. Source data is provided at the end of the Supplementary information file.

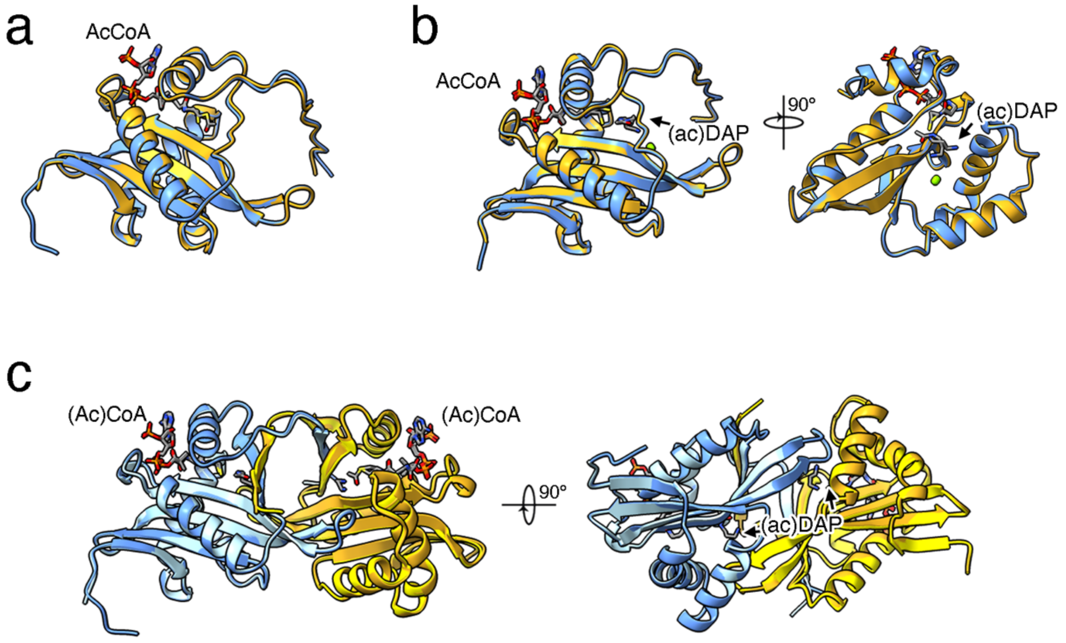

Supplementary Fig. S9 | Differences between monomers in Dpa crystal structures. (a) Alignment of acCoA bound Dpa monomers shown in blue and yellow; *r.m.s.d.*= 0.402. (b) Alignment of 1,3-DAP and acetyl-1,3-DAP bound monomers; *r.m.s.d.* = 0.302. (c) Alignment of free and (ac)-1,3-DAP bound dimers shown in darker and lighter tones of blue and yellow; *r.m.s.d.* = 0.282.

Supplementary Table S1 | X-ray data collection and processing.
The $CC_{1/2}$ criterion was used to determine the resolution range. Values for the outer shell are given in parentheses.

Sample	DpA	DpA-1,3DAP complex
Diffraction source	Soleil PX2	Soleil PX2
Wavelength (Å)	0.98015	0.9801
Temperature (K)	100.0	100.0
Detector	Eiger-X 16M	Eiger-X 16M
Crystal-detector distance (mm)	189.6	216.0
Rotation range per image (°)	0.1	0.1
Exposure time per image (s)	0.01	0.01
Space group	$P2_1$	$P2_1$
a, *b*, *c* (Å)	43.3 69.3 55.8	42.9 68.1 55.7
α, β, γ (°)	90.0 109.1 90.0	90.0 109.1 90.0
Mosaicity (°)	0.2	0.2
Resolution range (Å)	69.30 – 1.59	41.66 - 1.85
Total N°. of reflections	261030 (13389)	114641 (8723)
N°. of unique reflections	37959 (2163)	25128 (2407)

Completeness	90.07 (51.65)	97.37 (92.52)
Redundancy	6.9 (6.6)	4.6 (3.6)
⟨$I/\sigma(I)$⟩	14.55 (1.72)	8.43 (0.70)
$CC_{1/2}$	0.996 (0.695)	0.996 (0.418)
R_{pim}	0.033 (0.394)	0.054 (0.670)
Overall B factor / Wilson plot (Å^2)	19.99	34.39
R-factor (%)	18.2 (27.8)	19.6 (41.1)
R_{free}-factor (%)	20.7 (26.6)	23.2 (48.9)
Ramachandran profile (%)		
Core	99.3	98.6
Allowed	0.7	1.4
Outliers	0.0	0.0
R.m.s. deviations		
Bond lengths (Å)	0.012	0.011
Bond angles (°)	1.56	1.53
Number of atoms	2942	2882
Macromolecules	2514	2521
Solvent	307	236
Other	121	125
B-factors (Å2)		
All atoms	25.0	36.1
Macromolecules	23.3	35.0
Solvent atoms	38.9	46.7
Other atoms	25.2	39.5
PDB ID	8A9O	8A9N

Supplementary Table S2 | Primers used in the study.

Primer name	Sequence 5’-3’	Purpose	Reference
5FR_F	CAACAGTTATTTGCAGATCGT	Amplification of upstream region of *dpa*	This work
5FR_R	ACTCACTGTCTCTAATAAACCTAGGCGCTCCTTGTATTTATG		
3FR_F	GGTTTATTAGAGACAGTGAGT	Amplification of downstream region of *dpa*	This work
3FR_R	GTCGAGGCATTTCTGTCCTAGGTGATCTTACCCACTACA		
F_Dpa_AcORI_Ptac	TATTGCATGCTTGTAAGACGAGCGA	Amplification of *dpa* gene for complementation plasmid	This work

R_Dpa_AcORI_Ptac	GATGGTACCGGGATTGGGTATATTTGTCG		
F-hisDpa-Eco	GATCGAATTCATGCACCACCACCACCACCACATGATTGTAAGACGAGCG	Amplification of *A. baumannii dpa* gene for protein expression	This work
R-Dpa-Pst	GATCCTGCAGTTATTTAAGAAAACAATGAAATGT		
F-SpeG-Eco	GATCGAATTCATGCACCACCACCACCACCACATGCCAAGCGCCCACAGTGTTA	Amplification of *E. coli* speG gene for protein expression	This work
R-SpeG-Pst	GATCCTGCAGCTATTGTGCGGTCGGCTTCAGG		
F-abHpa2-Eco	GATCGAATTCATGCACCACCACCACCACCACATGTATAAAGTTATAGCGGGTAG	Amplification of *A. baumannii* hpa2 gene for protein expression	This work
R-abHpa2-Pst	GATCCTGCAGTCAACTTAACGCTAAAGTCATATCAA		
F-Aac6p-Eco	GATCGAATTCATGCACCACCACCACCACCACATGGACATCAGGCAAATGAACA	Amplification of *S. enterica* aac(6′)-Iy gene for protein expression	This work
R-Aac6p-Pst	GATCCTGCAGTCAACAACGCTTTCGGTAGAA		
F-Dpa- Y128F	TTCGAAAAAATGGGGTTTATTAGAG	Y128F mutation in Dpa	This work
R-Dpa- Y128F	GAGGCGGTGAGATTCATGA		
dpa_qF	TAGATGATGTATATGTTACTCC	qPCR	This work
dpa_qR	GGGTTTCTAGGCTAATACGT		
rpoB_qF	CGATTCGTACAGAACATTCTT	qPCR (housekeeping gene)	[1]

rpoB_qR	TAAAGCAGCATTGCCAGAATA		
tuf_qF	GAAGCGAAAGATTACTCACAA	qPCR	1
tuf_qR	CAGTGATCATGTTTTAACGTA		

Supplementary Table S3 | Amino acid sequences of acetyltransferases used in this study.

Host	Protein	Protein sequence
A. baumannii	His-Dpa	MHHHHHHMIVRRATYEDLSQLAVLFDEYRQFYGASSNLEESH HFLKQRFENKESVFFIHIKDEKITGFVLLYLGFSSVACSTYYILD DVYVTPLFRRQGSAKQLIDTAILFAKQENALRISLETQSNNHES HRLYEKMGFIRDSEFQTFHCFLK
E. coli	His-SpeG	MHHHHHHMPSAHSVKLRPLEREDLRYVHQLDNNASVMRYWF EEPYEAFVELSDLYDKHIHDQSERRFVVECDGEKAGLVELVEIN HVHRRAEFQIIISPEYQGKGLATRAAKLAMDYGFTVLNLYKLY LIVDKENEKAIHIYRKLGFSVEGELMHEFFINGQYRNAIRMCIFQ HQYLAEHKTPGQTLLKPTAQ
S. enterica	His-Aac(6')	MHHHHHHMDIRQMNRTHLDHWRGLRKQLWPGHPDDAHLAD GEEILQADHLVSFIAMADGVAIGFADASIRHDYVNGCDSSPVVF LEGIFVLPSFRQRGVAKQLIAAVQRWGTNKGCREMASDTSPEN TISQKVHQALGFEETERVIFYRKRC
A. baumannii	His-Hpa2	MHHHHHHMYKVIAGSWTQFEEDAKYIREQVFIQEQGIEPKDE WDDFDSTAVHFMVYDKEQPIATARLLPQHSVGRVAVLMLYRK QGIGKILMQHIIEYARQHKLPYLKLSAQTYVTAFYEALGFKVQ GEVYQDCGIPHIDMTLALS

Supplementary Methods

Antibiotic resistance tests.

For antbiotic resistance tests, cultures of DJ624Δara cells transformed with pKK223.3 vectors carrying different acetyltransferase enzymes were grown to optical density of A_{600} = 0.5, induced with 0.5 mM IPTG for 2 hours and a quantity of 0.5 OD unit of cells was spread on the solid LB agar containing 0.5 mM IPTG. Sterile Whatmann discs soaked in different concentrations of antibiotics were placed on the agar with bacteria and plates were incubated overnight revealing zones of growth inhibition. Amount equal to 0.1 OD unit of cells used for these experiments were boiled in protein loading buffer, migrated on 15% SDS-PAGE gel, proteins were transferred onto PVDF membrane, and expression of enzymes weas confirmed by western-blot with antibodies against his-tag (Sigma, ref. no. H1029, diluted 1:5000) followed by recognition with goat anti-mouse IgG-Alkaline Phosphatase antibodies (Sigma, A3562, diluted 1:5000) and detection with BCIP/NBT chromogenic substrate (Roche).

Supplementary References

1. Armalytė, J., Jurėnas, D., Krasauskas, R., Čepauskas, A. & Sužiedėlienė, E. The higBA Toxin-Antitoxin Module From the Opportunistic Pathogen Acinetobacter baumannii - Regulation, Activity, and Evolution. *Front Microbiol* **9**, 732 (2018).

Supplementary Source Data.

	Supplementary Figure S2a. Twitching motility											
	condition											
	WT	Δdpa	WT +DAP	Δdpa +DAP	WT PUT	Δdpa PUT	WT CAD	Δdpa CAD	WT SPD	Δdpa SPD	WT SPM	Δdpa SPM
	0.68	1.87	1.24	0.74	1.829	2.5	2.033	3.297	1.785	1.932	1.063	1.343
	1.21	2.01	1.33	0.98	1.252	2.683	2.222	2.248	1.239	1.651	0.942	1.258
	1.14	3.33	1.61	1.51	1.645	2.864	1.754	2.207	1.916	2.434	1.267	1.473
	1.75	2.20	1.69	1.51	2.009	3.444	0.947	2.807	1.882	2.601	0.957	0.93
	1.31	3.58	1.45	0.95								
	1.155	2.863	2.873	1.854								
	2.415	2.822	2.157	1.16								
	2.017	3.342	2.751	1.613								
area	1.727	3.067	3.008	1.993								

	Supplementary Figure S2b. Biofilm formation											
	condition											
	WT	Δdpa	WT +DAP	Δdpa +DAP	WT PUT	Δdpa PUT	WT CAD	Δdpa CAD	WT SPD	Δdpa SPD	WT SPM	Δdpa SPM
	4.1442682	1.83035672	4.47494198	1.29200025	4.16247868	1.68693533	3.87947314	3.49770757	4.83572185	2.15234544	4.4340171	1.69093651
	4.6313483	1.495617	5.20078991	1.17698387	4.63626979	1.09033186	4.83734775	2.47414235	4.64174068	1.53865329	4.16871309	1.17047403
	3.51391879	2.12134291	2.9201305	2.53212873	3.32132712	1.91638488	3.44512704	1.86421387	5.12065639	2.49397309	4.07657786	2.272399
OD 580/600 nm	5.07853736	1.49116318	4.71617671	1.81397438	4.93093454	1.36592535	4.10154792	1.5015702	5.17743446	1.7715699	3.34942302	1.91985843

Supplementary Figure S3c								
Raw data for enzyme kinetics								
	mM, 1,3DAP	time, min						
replicate 1		1	2	5	10	15	30	45	60
	2	0.11734287	0.00365141	0.09126163	2.37113713	7.43116159	16.258206	22.5722516	29.2268739
	5	0.46922364	3.29785225	8.69437934	17.1959941	18.2424669	29.0552508	36.3369892	51.1157017
	7.5	1.77038165	2.74848844	11.5140404	17.659562	21.3386015	35.0666868	52.5655289	62.4198547
	10	2.0416458	3.39746464	10.184943	16.7906474	23.6117224	44.5122529	61.8599106	68.2686592
	12.5	4.21830951	13.1317563	19.9004712	37.7616419	48.3796367	76.1377751	88.5912513	93.7642275
	15	8.66048126	13.431821	19.7918917	35.8269947	54.4572624	78.5773493	89.4298123	95.3317603
replicate 2		1	2	5	10	15	30	45	60
	2	0.48805022	2.2549676	5.09624888	7.99700281	10.6240178	20.3711833	24.2896076	31.1238713
	5	3.41762644	4.77755545	9.98168089	16.8561664	19.6376214	32.7310235	41.1868733	48.29908
	7.5	1.92515518	2.41218022	10.2402794	17.4599222	24.4688541	37.3214912	51.2688215	61.98171
	10	3.19015421	4.55222948	10.46484	17.0965487	24.4250707	46.2765253	63.0828252	68.0155776
	12.5	5.61000749	13.6200346	18.8258257	38.0225894	51.1487025	78.3546755	90.6770658	94.340592
	15	10.5530897	15.9139498	23.2725994	39.5631235	56.78919	81.3371711	90.9108846	96.2413868
replicate 3		1	2	5	10	15	30	45	60
	2	1.32468208	1.83314172	3.80187289	6.81518291	9.67762334	21.4200832	24.1346115	31.9674018
	5	2.9459743	4.48441325	10.9167158	17.083421	19.3760488	32.4879057	41.6469779	51.6441552
	7.5	2.38407451	4.03475508	10.3805281	18.6503525	21.8767212	36.5708233	52.0734231	62.2835075
	10	3.492194	3.635445	12.1912126	18.5136678	25.2126476	45.9871303	63.5894441	69.1502639
	12.5	6.91728998	13.9315337	19.5843059	41.0839354	51.4790504	78.8135847	90.9406659	94.6525299
	15	11.8514298	17.8085052	24.4826223	39.3825173	57.0124546	81.6123227	90.9376717	96.3997087

Supplementary Figure S4c												
Acetylation of antibiotics by DTNB method												
substrate	enzyme											
	AbDpa	AbDpa	AbDpa	EcSpeG	EcSpeG	EcSpeG	AbHpa2	AbHpa2	AbHpa2	SeAac(6')-Iy	SeAac(6')-Iy	SeAac(6')-Iy
STRP	0.00482331	0.00330272	0.00281613	0.02769296	0.02720637	0.02787543	0.00330272	0.00281613	0.00214707	0.0071346	0.00409342	0.00415425
KAN	-0.0084971	-0.0024147	-0.0038136	0.01698802	0.0218539	0.02221884	-0.0093486	-0.0049085	-0.0028405	0.34586096	0.39537133	0.43630558
GENT	-0.0045435	-0.0098352	-0.0046043	0.01966425	0.01005413	0.01139225	-0.0065507	-0.0102609	-0.0095919	0.39512803	0.38892403	0.46817712
AMIKA	0.00056566	0.0009306	-0.0004683	0.02422602	0.02349614	0.02361778	0.00232954	-0.0013807	-0.0007116	0.16272125	0.17281796	0.18553008
TOBRA	-0.006794	-0.0163433	-0.0153093	0.02343531	0.01431178	0.01224378	-0.0038745	-0.0087403	-0.0102001	0.28886929	0.35303814	0.36167508

Supplementary Figure S5e

Acetylation of amino acids by DTNB method

substrate	enzyme											
	AbDpa	AbDpa	AbDpa	AbHpa2	AbHpa2	AbHpa2	SeAac(6')-Iy	SeAac(6')-Iy	SeAac(6')-Iy	EcSpeG	EcSpeG	EcSpeG
Arg	0.00169179	0.00826087	0.00285696	0.1195035	0.14557749	0.1306361	0.00577074	0.00280578	0.00311016	0.03277968	0.03422856	0.0385436
His	0.00282327	0.00369269	0.00248257	0.07786398	0.07334602	0.07051902	0.00145638	0.00051786	0.00649397	0.01371802	0.02095236	0.02296506
Lys	0.00796703	0.00485361	0.00395809	0.02450991	0.02365709	0.02401236	0.00368572	0.00789478	0.0045956	0.01134168	0.00895228	0.00882292
Asp	0.00062284	0.00302683	0.000111	0.00733616	0.00288114	0.0003637	-0.0011532	-0.0010029	0.0008875	0.00028053	0.00040899	0.00028316
Glu	0.0074315	0.00883609	0.0079819	0.00573074	0.00708044	0.0077857	0.00749915	0.00383908	0.00955434	0.0100808	0.00804303	0.00854117
Ser	0.0037669	0.00946688	0.01077745	0.1002322	0.12861336	0.12422757	0.00263756	0.00705604	0.00466535	0.08236017	0.08234187	0.06402658
Thr	0.00553008	0.00385926	0.00294929	0.69368936	0.62690853	0.64827499	0.00064735	0.00227721	0.00232179	0.30134831	0.29227764	0.30703259
Asn	0.00840388	0.00844643	0.00891265	0.01137694	0.01172816	0.01057566	0.00476244	0.00671961	0.00604953	0.00783801	0.01392177	0.01000898
Gln	0.01190651	0.013574	0.00701953	0.00942108	0.01025059	0.01046339	0.00495506	0.00493202	0.00243648	0.01188273	0.01926508	0.01379379
Gly	0.00677855	0.00931192	0.00356725	0.01449159	0.01131224	0.00851551	0.00238036	0.00089003	0.00149106	0.02579958	0.00365538	0.01170028
Pro	0.00451039	0.00147415	0.01161939	0.00771057	0.00207697	0.0107598	0.00429943	0.00575574	0.00468414	0.00224844	0.01040894	0.01529322
Ala	0.00976813	0.00476831	0.00512589	0.00432359	0.00973954	0.00432941	0.00186008	0.0041466	0.0019542	0.01063442	0.00804844	0.01537167
Ile	0.00953588	0.01075953	0.00921045	0.01021472	0.00912811	0.01058168	0.00526384	0.00354812	0.00820606	0.01831893	0.01133747	0.0170214
Leu	0.00901468	0.01211177	0.00788119	0.00702262	0.01421907	0.00813191	0.00623136	0.00617227	0.01011801	0.01489416	0.01233182	0.01988711
Met	0.00454445	0.00824306	0.00589627	0.00870033	0.01204806	0.01110837	0.00208477	0.0014461	0.00406221	0.00839001	0.01700445	0.01668438
Phe	0.00819931	0.00505837	0.00668325	0.01010297	0.01837487	0.01868737	0.00183464	0.006466	0.00445541	0.01325403	0.01445412	0.0130202
Trp	0.0028106	0.01461203	0.01004278	0.21580552	0.42628457	0.4050816	0.00365534	0.00215496	0.00591789	0.0997347	0.09286092	0.10291514
Tyr	0.0115506	0.01183584	0.01325752	0.06726433	0.20654531	0.2123586	0.00895608	0.00707691	0.01438933	0.04715364	0.03425317	0.0424683
Val	0.00419011	-0.0019433	-0.0037194	0.01069275	0.00044864	-0.0010078	-0.0023811	-0.0030523	-0.0030735	0.00760592	0.00799457	0.00975159
beta-Ala	-0.0010239	-0.0008292	0.00464794	0.00835248	0.00746463	0.00841244	0.00168846	0.00392891	-0.0034437	0.01105343	0.01477645	0.01193931
Agmt	0.01619912	0.01475953	0.01199687	0.03323548	0.05158979	0.04765952	0.00723435	0.01270617	0.00740883	0.40623155	0.42754547	0.38861213

Supplementary Figure S8.

1,3-DAP kinetics measured by DTNB method

time, min	Dpa WT	Dpa WT	Dpa WT	Dpa WT+EDT	Dpa WT+EDT	Dpa WT+EDT	DpaY128F	DpaY128F	DpaY128F
1	7.9071E-05	0.00038319	0.00044401	0.00032236	0.00044401	0.00032236	0.00044401	0.00056566	0.00044401
2	0.01218296	0.00956754	0.00914178	0.00865519	0.0084119	0.00804696	0.00749954	0.00603978	0.00658719
3	0.02319202	0.01869108	0.01777872	0.01613649	0.01577155	0.01510249	0.01206131	0.00968919	0.01066237
4	0.03468767	0.02574661	0.02446931	0.02307037	0.02252296	0.02191473	0.01492002	0.01200049	0.01339943
5	0.04527097	0.03322791	0.03182896	0.02927438	0.02878779	0.02817955	0.01747461	0.01419013	0.01589319
6	0.05524603	0.04101332	0.0388845	0.03541755	0.03517426	0.03468767	0.01936014	0.0156499	0.01783955
7	0.06509945	0.04825132	0.04557509	0.04125661	0.04119579	0.04143909	0.02088073	0.01692719	0.01972508
8	0.07458792	0.05597591	0.0523265	0.04727815	0.04721732	0.04855544	0.0220972	0.01777872	0.02112402
9	0.08389392	0.06363968	0.0595645	0.05366462	0.05342132	0.05585427	0.02331367	0.0187519	0.02282708
10	0.09368652	0.07130345	0.06649839	0.06011192	0.05986862	0.06278815	0.0239219	0.01905602	0.02380026
11	0.10341828	0.07921051	0.07373639	0.06649839	0.06680251	0.07026945	0.0251992	0.02009002	0.02538167
12	0.1130284	0.08705675	0.08127851	0.0734931	0.07373639	0.07781157	0.02611155	0.02075908	0.02696308
13	0.12349006	0.09472052	0.0882124	0.08042698	0.08085275	0.08559698	0.02665896	0.02094155	0.02799708
14	0.13334347	0.10281005	0.09581534	0.08730004	0.0882124	0.09259169	0.02763214	0.02179308	0.02939602
15	0.14398759	0.10980476	0.10238428	0.0944164	0.09526793	0.10007299	0.02836202	0.02221884	0.03073414
16	0.15414512	0.11759017	0.10968311	0.10122864	0.10220181	0.10743264	0.02945685	0.02294873	0.03219391
17	0.16448513	0.12494982	0.11673864	0.10816252	0.10919652	0.11473146	0.03030838	0.02361778	0.03341038
18	0.17427772	0.13218782	0.12336841	0.11485311	0.11606958	0.12172617	0.03097743	0.0239219	0.03462685
19	0.18364455	0.13948665	0.13048476	0.12105711	0.12215194	0.12829512	0.03182896	0.02459096	0.03608661
20	0.19191655	0.14623806	0.13693206	0.12689617	0.12835594	0.13413418	0.03268049	0.0251992	0.03724226
21	0.20012773	0.15341524	0.14380512	0.13224865	0.13383006	0.14015571	0.03298461	0.02544249	0.03827626
22	0.20797397	0.1602883	0.15043489	0.13772277	0.13893924	0.14550818	0.03401861	0.02611155	0.03961438
23	0.21612432	0.16728301	0.15694301	0.14337936	0.14502159	0.15092148	0.0345052	0.02647649	0.04040508
24	0.22421386	0.1739736	0.16345113	0.14915759	0.15055654	0.15676054	0.03529591	0.02690226	0.04162156
25	0.23169515	0.18090749	0.1700809	0.15475336	0.15627395	0.1623563	0.03632991	0.02769296	0.04308132
26	0.23881151	0.1879022	0.17689313	0.16059242	0.16223466	0.16789125	0.03687732	0.02799708	0.04399367
27	0.24750928	0.19416702	0.18297549	0.16630984	0.16801289	0.17385196	0.03742473	0.02824037	0.04502767
28	0.25462563	0.20128338	0.18966608	0.17208807	0.17360866	0.17920443	0.03839791	0.02921355	0.04636579
29	0.26186363	0.20718326	0.19556596	0.17786631	0.17932608	0.18486102	0.03912779	0.02957849	0.04764309
30	0.26928411	0.21356973	0.20164832	0.18370537	0.18516514	0.19057843	0.03943191	0.02976096	0.04812968
31	0.27627882	0.22038197	0.20821726	0.18936196	0.19088255	0.19587008	0.04022261	0.03024755	0.04928532
32	0.28260446	0.22646433	0.21423879	0.19483608	0.19653914	0.20104008	0.0407092	0.03055167	0.05013685
33	0.28874764	0.23254668	0.22007785	0.20037102	0.20243902	0.20602761	0.04131744	0.03097743	0.05117085
34	0.29501247	0.23808163	0.2256128	0.20596679	0.20785232	0.21101515	0.04168238	0.03115991	0.05214403
35	0.30018247	0.24416398	0.23151268	0.21144091	0.21308315	0.21600268	0.04265556	0.03195061	0.0533605
36	0.3054133	0.24982057	0.23674351	0.21661091	0.2181315	0.22062527	0.0430205	0.03219391	0.05402956
37	0.30973177	0.2550514	0.24209598	0.22165927	0.2233015	0.22494374	0.04362873	0.0324372	0.05494191
38	0.31392859	0.26040387	0.24726598	0.22682927	0.22822821	0.22859315	0.04435862	0.03310626	0.05615838
39	0.31648318	0.26502646	0.25188857	0.23133021	0.23291162	0.23236421	0.0448452	0.03328873	0.05694909
40	0.31873365	0.2697707	0.25638951	0.23558786	0.2372301	0.23558786	0.04514932	0.03353202	0.05743568
41	0.32134907	0.2736634	0.2602214	0.23996716	0.24191351	0.23856821	0.04581838	0.03389696	0.05865215
42	0.32226142	0.27743446	0.26380999	0.2444681	0.2455021	0.24100116	0.04648744	0.03444438	0.05974697
43	0.32359954	0.28053646	0.26709446	0.24836081	0.24945563	0.24367739	0.04697403	0.03493097	0.06053768
44	0.32451189	0.28315188	0.26922328	0.25219269	0.25346998	0.24483304	0.04685238	0.03462685	0.06041603
45	0.32445107	0.28588894	0.27226446	0.25541634	0.25657198	0.24659692	0.04733897	0.03468767	0.06138921
46	0.32499848	0.28789611	0.27408917	0.25870081	0.25967399	0.24726598	0.04764309	0.03499179	0.06205827
47	0.32390366	0.28953835	0.27591387	0.26137705	0.26198528	0.24836081	0.04800803	0.03523508	0.06278815
48	0.32372118	0.29075482	0.27706952	0.26380999	0.26478316	0.24884739	0.04849462	0.03560002	0.06376133
49	0.32256554	0.29166717	0.27761693	0.26606046	0.26672952	0.24927316	0.04861626	0.0355392	0.06400462
50	0.32177483	0.29209294	0.27792105	0.26776352	0.26806764	0.24890822	0.04916368	0.03584332	0.06479533
51	0.3198893	0.29203211	0.2780427	0.26922328	0.2697707	0.24878657	0.04958944	0.03620826	0.06552521
52	0.31903777	0.29166717	0.27786023	0.27031811	0.27092634	0.24823916	0.04995438	0.03651238	0.06613345
53	0.31703059	0.29154553	0.27749529	0.27104799	0.2718387	0.24708351	0.05013685	0.03645155	0.06668086
54	0.31593577	0.29087647	0.27664376	0.27129128	0.27171705	0.24623198	0.05050179	0.0365732	0.06734992
55	0.31398942	0.29020741	0.27609634	0.27189952	0.27196034	0.24538045	0.05104921	0.03705979	0.06832309
56	0.31192142	0.288626	0.27463658	0.2718387	0.2718387	0.24398151	0.05117085	0.03705979	0.06856639
57	0.31009671	0.28789611	0.27360258	0.27141293	0.27165623	0.24258257	0.05177909	0.03730308	0.06929627
58	0.30766377	0.28613223	0.27171705	0.27104799	0.27104799	0.24154857	0.05190074	0.03730308	0.06972204
59	0.30608236	0.28467247	0.27037893	0.2705614	0.27007481	0.23923727	0.05250897	0.03772885	0.07063439
60	0.30383188	0.28303023	0.26861505	0.2695274	0.26964905	0.23765586	0.05275227	0.03803297	0.0711818

Figure S1

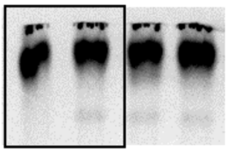

Figure S3 a

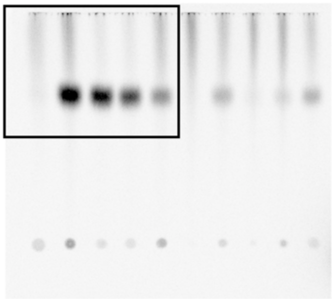

Figure S3 b

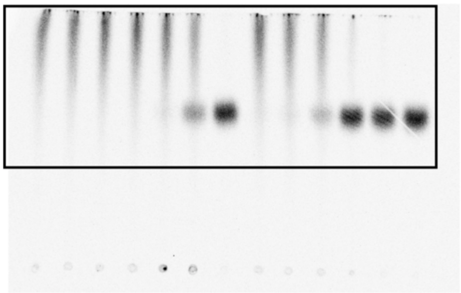

Figure S4 b

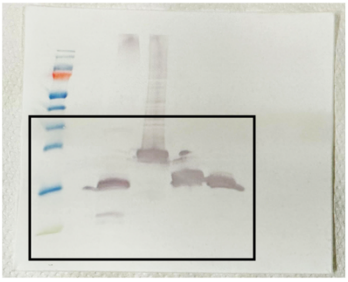

Figure S7 l

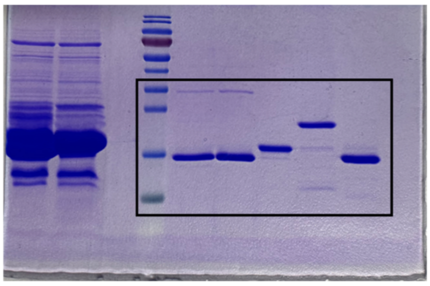

Uncropped gels, western blots and autoradiography scans of supplementary figures. Area used for preparing the figures is boxed. Source data for main figures is provided in a separate source data file.
