# Supplementary material for: A polyamine acetyltransferase regulates the motility and biofilm formation of Acinetobacter baumannii
Source: Nat Commun. 2023 Jun 14;14:3531. doi: 10.1038/s41467-023-39316-5 (PMC10267138; doi:10.1038/s41467-023-39316-5)
Supplement: Supplementary file 3 — Reporting Summary [file 41467_2023_39316_MOESM3_ESM.pdf]

nature portfolio

Corresponding author(s): Dukas Jurėnas, Abel Garcia-Pino

Last updated by author(s): May 25, 2023

# Reporting Summary

Nature Portfolio wishes to improve the reproducibility of the work that we publish. This form provides structure for consistency and transparency in reporting. For further information on Nature Portfolio policies, see our Editorial Policies and the Editorial Policy Checklist.

## Statistics

For all statistical analyses, confirm that the following items are present in the figure legend, table legend, main text, or Methods section.

| n/a | Confirmed | |
|---|---|---|
| ☐ | ☒ | The exact sample size (*n*) for each experimental group/condition, given as a discrete number and unit of measurement |
| ☐ | ☒ | A statement on whether measurements were taken from distinct samples or whether the same sample was measured repeatedly |
| ☐ | ☒ | The statistical test(s) used AND whether they are one- or two-sided<br>*Only common tests should be described solely by name; describe more complex techniques in the Methods section.* |
| ☒ | ☐ | A description of all covariates tested |
| ☒ | ☐ | A description of any assumptions or corrections, such as tests of normality and adjustment for multiple comparisons |
| ☐ | ☒ | A full description of the statistical parameters including central tendency (e.g. means) or other basic estimates (e.g. regression coefficient) AND variation (e.g. standard deviation) or associated estimates of uncertainty (e.g. confidence intervals) |
| ☐ | ☒ | For null hypothesis testing, the test statistic (e.g. *F*, *t*, *r*) with confidence intervals, effect sizes, degrees of freedom and *P* value noted<br>*Give P values as exact values whenever suitable.* |
| ☒ | ☐ | For Bayesian analysis, information on the choice of priors and Markov chain Monte Carlo settings |
| ☒ | ☐ | For hierarchical and complex designs, identification of the appropriate level for tests and full reporting of outcomes |
| ☒ | ☐ | Estimates of effect sizes (e.g. Cohen's *d*, Pearson's *r*), indicating how they were calculated |

*Our web collection on statistics for biologists contains articles on many of the points above.*

## Software and code

Policy information about availability of computer code

| | |
|---|---|
| Data collection | SoftMax Pro 6.3 |
| Data analysis | XDS Version January 26, 2018 (BUILT=20180126), XSCALE (part of the XDS package), AIMLESS 0.5.24, PHENIX 1.18, POINTLESS 1.10.21, MATTHEWS_COEF (part of the CCP4 suite 7.0), HKL2MAP suite 0.3.i-beta, ArpWarp 7.1, Buster/TNT 2.10.4, Coot 0.8, ShelxD 2016/1, ShelxE 2016/1, ImageJ 1.53k |

For manuscripts utilizing custom algorithms or software that are central to the research but not yet described in published literature, software must be made available to editors and reviewers. We strongly encourage code deposition in a community repository (e.g. GitHub). See the Nature Portfolio guidelines for submitting code & software for further information.

## Data

Policy information about availability of data

All manuscripts must include a data availability statement. This statement should provide the following information, where applicable:

- Accession codes, unique identifiers, or web links for publicly available datasets
- A description of any restrictions on data availability
- For clinical datasets or third party data, please ensure that the statement adheres to our policy

The final atomic model and coordinates of Dpa and of the Dpa in complex with the 1,3-DAP have been deposited to the Protein Data Bank (PDB) under the accession codes 8A9O and 8A9N respectively. Previously reported structures referenced in this study are available in the PDB under accession codes: 4R9M, 3BJ7,

1S5K, 1QSO, 1TIQ, 4R87, 1QSM, 3BJ8, 5JPH, 4JLY, 2B5G. Gene sequence can be accessed in GenBank (ID: OQ718427). Source data, uncropped gels and Western-blots are provided in Source Data file and in Supplementary Information.

## Research involving human participants, their data, or biological material

Policy information about studies with human participants or human data. See also policy information about sex, gender (identity/presentation), and sexual orientation and race, ethnicity and racism.

| | |
|---|---|
| Reporting on sex and gender | n/a |
| Reporting on race, ethnicity, or other socially relevant groupings | n/a |
| Population characteristics | n/a |
| Recruitment | n/a |
| Ethics oversight | n/a |

Note that full information on the approval of the study protocol must also be provided in the manuscript.

# Field-specific reporting

Please select the one below that is the best fit for your research. If you are not sure, read the appropriate sections before making your selection.

☒ Life sciences ☐ Behavioural & social sciences ☐ Ecological, evolutionary & environmental sciences

For a reference copy of the document with all sections, see nature.com/documents/nr-reporting-summary-flat.pdf

# Life sciences study design

All studies must disclose on these points even when the disclosure is negative.

| | |
|---|---|
| Sample size | This work does not include sampling large populations. For in vivo experiments, as accepted in the biological studies, at least three independent biological replicas were performed, in experiments with high variability the sample size was doubled. For biochemical tests, at least three replicates were performed, where each replicate was assembled and measured independently. |
| Data exclusions | No measured data was excluded. |
| Replication | Reproducibility was ensured by repeated measurements (at least three) following the strict procedures of the protocol and all the obtained data from all replicates was represented. Experiments with polyamines added in the medium that have high variability are cautioned in text. |
| Randomization | For bacterial inoculations random colonies were selected to start overnight cultures. For biochemical studies randomization is not relevant. |
| Blinding | investigators were aware of sample identities, but not aware of hypotheses or had no a priori hypothesis of the outcome of the experiments. |

# Reporting for specific materials, systems and methods

We require information from authors about some types of materials, experimental systems and methods used in many studies. Here, indicate whether each material, system or method listed is relevant to your study. If you are not sure if a list item applies to your research, read the appropriate section before selecting a response.

### Materials & experimental systems

| n/a | Involved in the study | |
|---|---|---|
| ☐ | ☒ | Antibodies |
| ☐ | ☒ | Eukaryotic cell lines |
| ☒ | ☐ | Palaeontology and archaeology |
| ☒ | ☐ | Animals and other organisms |
| ☒ | ☐ | Clinical data |
| ☒ | ☐ | Dual use research of concern |
| ☒ | ☐ | Plants |

### Methods

| n/a | Involved in the study | |
|---|---|---|
| ☒ | ☐ | ChIP-seq |
| ☒ | ☐ | Flow cytometry |
| ☒ | ☐ | MRI-based neuroimaging |

## Antibodies

| | |
|---|---|
| Antibodies used | Monoclonal mouse Anti-polyHistidine, Clone HIS-1 (Sigma, H1029) and goat Anti-Mouse IgG–Alkaline Phosphatase (Sigma, A3562) |
| Validation | Primary antibodies (Sigma, H1029) were validated by manufacturer to preferentially recognize native and denatured-reduced forms of synthetic polyhistidine or N-terminally tagged polyhistidine fusion proteins expressed by prokaryotic pET, pRSET, and pTrc expression vectors. Antibody was reactive in immunoblotting, dot blot, immunofluorescent staining of cultured cells and ELISA. Antibody is routinely used in our laboratory. To ensure specificity, control with empty expression vector was included. |

## Eukaryotic cell lines

Policy information about cell lines and Sex and Gender in Research

| | |
|---|---|
| Cell line source(s) | LL/2 (LLC1) Lewis lung carcinoma cell line was acquired from ATCC (ref, CRL-1624) |
| Authentication | Cell line was not authenticated |
| Mycoplasma contamination | Cell line was not tested for mycoplasma contamination |
| Commonly misidentified lines (See ICLAC register) | not used |
